# Radiotherapy for stage IIA rectal cancer may not benefit all

**DOI:** 10.18632/oncotarget.19683

**Published:** 2017-07-29

**Authors:** Xiang Hu, Ya-Qi Li, Qing-Guo Li, Yan-Lei Ma, Jun-Jie Peng, San-Jun Cai

**Affiliations:** ^1^ Department of Colorectal Surgery, Fudan University Shanghai Cancer Center, Shanghai, 20032, China; ^2^ Department of Oncology, Shanghai Medical College, Fudan University, Shanghai 200032, China

**Keywords:** rectal cancer, stage IIA, radiotherapy, outcomes

## Abstract

This study sought to determine whether additional radiotherapy is necessary in patients after optimal surgery for stage IIA rectal cancer and how the different covariates influence the efficacy of radiotherapy. The first primary rectal cancer was identified from the 1988–December 2013 Surveillance, Epidemiology and End Results database. We identified 13647 patients with IIA rectal cancer, in which 39.6% received neo-adjuvant radiotherapy and in another 14.96% patients the adjuvant radiotherapy were performed. Neo-adjuvant or adjuvant radiotherapy group had better survival with 10-Year cancer-specific survival estimates as 75.1% and 73.8% compared to 68.4% of no radiotherapy group (*P* < 0.01). Adjusted hazard ratio (HR) demonstrated neo-adjuvant and adjuvant radiotherapy (HR: 0.814 and 0.848) were all associated with significantly decreased risk for cancer death. However, radiotherapy did not seem to yield the same survival benefit in selected population. Adjusted stratified analysis demonstrated patients with increasing age, relative large tumor size, and more retrieved regional lymph nodes had no additional benefit for cancer specific survival based on radiation use. In conclusions, unselected patients with stage IIA rectal cancer receiving radiotherapy experienced better survival in comparison to patients without radiation. However, additional radiotherapy is not beneficial for all.

## INTRODUCTION

Rectal cancer is one of the most common cancers with high morbidity in the worldwide [1]. Substantially improvement in the management of locally advanced rectal cancer has been achieved during the last decade, through the use of additional chemoradiotherapy [2] and the introduction of total mesorectal excision (TME) [3]. Although local recurrence after curative resection of rectal cancer has been reduced significantly [4], local failure was also estimated up to 29% [5], which affected the prognosis with reduced disease-specific survival. So additional radiation therapy (RT) consequently becomes the standard care for stage II or node-positive rectal cancer because of the confirmed oncologic benefit [6]. However, this standard adjuvant treatment was established before the era of combined-modality therapy. Therefore, the advent and success of modern regimen called into question the oncologic benefit of adjuvant RT in lower risk patients specifically those at stage IIA. With regard to the role of RT for preventing local failure and mortality, some controversy persists. Neo-Adjuvant RT was definitively established as standard of care for stage II or III rectal cancer in German Rectal Cancer Study and National Surgical Adjuvant Breast and Bowel Project (NSABP) [2, 7]. However, the Dutch Colorectal Study Group showed that there was no significant increase in overall survival on the basis of radiation, and only improvement in rates of local control [8]. What is more, it has shown that additional postoperative radiotherapy did not alter local recurrence or survival after TME in patients with stage IIA rectal cancer [9]. Based on those retrospective studies [10, 11], it was indicated that radiotherapy could be omitted in early-stage rectal cancer patients if the optimal surgery was performed because of comparable overall local recurrence and survival between groups. Moreover, some patients receiving radiotherapy may be disadvantaged in terms of radiation related complication and cost effectiveness, particularly in stage IIA disease. It is important to note that these data are of heterogeneity in nature in result of small sample size, hence, there is great variation in outcome for patients with stage IIA cancers, and the outcome is hard to interpret. We postulate that stage IIA rectal cancers should be analyzed and treated separately from other rectal cancers. Therefore, the benefit of additional radiotherapy should be analyzed and stratified by gender, age at diagnosis, histological grade, tumor size, regional nodes examined and so on to shed more light on this particular subset. The purpose of this study was, therefore, to determine whether additional radiotherapy is necessary in patients after optimal surgery for stage IIA rectal cancer and how the different covariates influence the efficacy of radiation therapy.

## RESULTS

### Patient characteristics

There were a total of 13647 records in the Surveillance, Epidemiology and End Results database (SEER) from 1988-2013, meeting the inclusion criteria available for analysis. Of the included patients in this study, 39.6% received neo-adjuvant radiation therapy and the adjuvant radiation therapy was performed in another 14.96% patients, while the remaining 45.44% did not receive radiation. In this cohort, the median age was 964 years old (interquartile range, 55 to 74 year-old), the median tumor size was 44 mm (range, 30 to 60 mm), and the median number of retrieved regional lymph nodes was 12 (range, 5 to 17 nodes). The patients demographic and tumor characteristics in this study are summarized in Table 1.

**Table 1:** Description of the study population, public use SEER database 1988-2013 stratified by additional radiation use

Variables	No radiation (6204)	Adjuvant RT (2041)	Neo-adjuvant RT (5402)	*P* value
**Gender**				
** Male**	3444(55.5%)	1274(62.4%)	3445(63.8%)	< 0.01
** Female**	2760(44.5%)	767(37.6%)	1957(36.2%)	
**Diagnosis year**				
** 1988–1997**	1756(28.3%)	561(27.5%)	1300(24.1%)	0.42
** 1998–2007**	2060(33.2%)	696(34.1%)	1961(36.3%)	
** 2008–2013**	2388(38.5%)	784(38.4%)	2141(39.6%)	
**Median follow-up, M**	38	56	44	< 0.01
**Age, Y**				< 0.01
** < 65**	2622(42.3%)	1263(61.9%)	3422(63.3%)	
** 65–84**	2877(46.4%)	741(36.3%)	1880(34.8%)	
** > 85**	705(11.3%)	37(1.8%)	100(1.9%)	
**Race**				0.95
** White**	5169(83.3%)	1653(80.9%)	4321(80%)	
** Black**	428(6.9%)	127(6.2%)	340(6.3%)	
** Other**	607(9.8%)	261(12.9%)	741(13.7%)	
**Marital status**				0.72
** Married/partnered**	3027(48.8%)	1142(55.9%)	2809(52%)	
** Un-partnered**	2916(47%)	857(42%)	2484(46%)	
**Grade**				< 0.01
** well**	454(7.3%)	163(8%)	363(6.7%)	
** moderate**	4639(74.8%)	1569(76.9%)	3823(70.8%)	
** poor**	614(9.9%)	222(10.9%)	510(9.4%)	
** undifferentiated**	45(0.7%)	22(1.1%)	43(0.8%)	
**Tumor size (mm)**				< 0.01
** < 50**	3402(54.8%)	1183(57.9%)	3164(58.6%)	
** 50–100**	1910(30.8%)	661(32.4%)	908(16.8%)	
** > 100**	93(1.5%)	35(1.7%)	72(1.3%)	
** Mean**	49.1 ± 22.9	48.64 ± 26.2	42.8 ± 26.5	
**Lymph node examined**				< 0.01
** < 15**	3925(63.3%)	1232(60.4%)	3941(72.9%)	
** ≥ 15**	2225(35.9%)	791(38.8%)	1414(262%)	
** Median**	12	13	11	
**Surgical type**				0.21
** Sphincter preservation**	3741(60.3%)	1188(58.2%)	3133(58%)	
** APR**	2364(38.1%)	829(40.6%)	2112(39.1%)	

RT indicates radiotherapy; APR, Abdominoperineal resection; M, month; Y, year.

### Radiation therapy and clinicopathologic Covariates

Patients undergoing radiation therapy were more in average younger group than those in elderly population (64.1% vs. 16.3%). Majority were the male in gender in all groups. And several other factors involved with use of radiation therapy are presented in Table 1. Comparing to patients not receiving radiation treatment, those undergoing neo-adjuvant and adjuvant radiotherapy had more common of poor pathological differentiation, larger size of carcinoma lesion and less retrieved lymph nodes, (*P* < 0.001, for all). In addition, patients who were separated were comparable to undergo radiation therapy relative to partnered ones.

### Overall and cause-specific survival

In this cohort, it demonstrated that mortality rate was 24.89% (*n* = 3397) in all. Of the patients who died from all causes, 64.7% (*n* = 2198) did not receive radiation therapy and 15.19% (*n* = 516) received adjuvant radiation, as well as 20.11% in the neo-adjuvant radiation treatment group. In terms of cancer specific motality, 2202 patients died from rectal cancer, of who, 53.4% (*n* = 1198) did not get any radiation therapy, 14.58% (*n* = 321) received adjuvant radiation and the remaining 683 victims were from neo-adjuvant radiation group (Table 2). Among the patients receiving neo-adjuvant radiation therapy (*n* = 5402) and adjuvant radiation (*n* = 2041), 81.4% and 72.7% were survived at the end of the follow-up period time, comparing to 64.6% of patients without radiation treatment. It was estimated that the 10-year cumulative overall survival was 61.8% and 59.5% in those who received neo-adjuvant radiation and adjuvant radiation therapy, while the survival rate was only 42.8% in those who did not. Additionally, the cumulative rectal cancer-specific survival was presented in the same pattern at 1, 3, 5, and 10 year regarding to radiation therapy (Table 3). Significance was demonstrated all the same in rectal cancer-specific survival at any time point. At 5-year follow-up, 84.3% and 83.9% patients receiving neo-adjuvant and adjuvant radiotherapy were alive comparing to 76.5% without any radiotherapy. In terms of 10-year cancer specific survival, 75.1% and 73.8% were estimated in neo-adjuvant and adjuvant radiation group, compared with 68.4% in patients receiving no radiation therapy. However, No differences in survival were seen between the group of neo- and adjuvant radiation.

**Table 2:** Cumulative overall and cancer-specific vital status

Variables	No radiation(6204)	Adjuvant RT(2041)	Neo-adjuvant RT(5402)	*P* value
**Over all status**				< 0.01
** Alive**	4007(64.6)	1526(74.7)	4397(81.4)	
** Died**	2197	515	1005	
**Cancer specific survival**				< 0.01
** Alive**	5007(80.7)	1720(84.3)	4720(87.4)	
** Died**	1197	321	683	

RT indicates radiotherapy.

**Table 3:** Year survival rates (1, 3, 5, and 10) by radiation status, public use SEER database 1988–2013

Survival	No radiation	Adjuvant RT	Neo-adjuvant RT
**Overall survival**			
**1 year rate**	88.5	97	96.6
**3- year rate**	73	86.7	88.1
**5- year rate**	62.6	77.1	78.5
**10-year rate**	42.8	59.5	61.8
**cancer-specific survival**			
**1 year rate**	93.2	98.4	97.9
**3- year rate**	82.9	90.8	91.6
**5- year rate**	76.5	83.9	84.3
**10-year rate**	68.4	73.8	75.1

RT indicates radiotherapy.

### Survival analysis and the impact of radiation therapy

**Figure 1:**
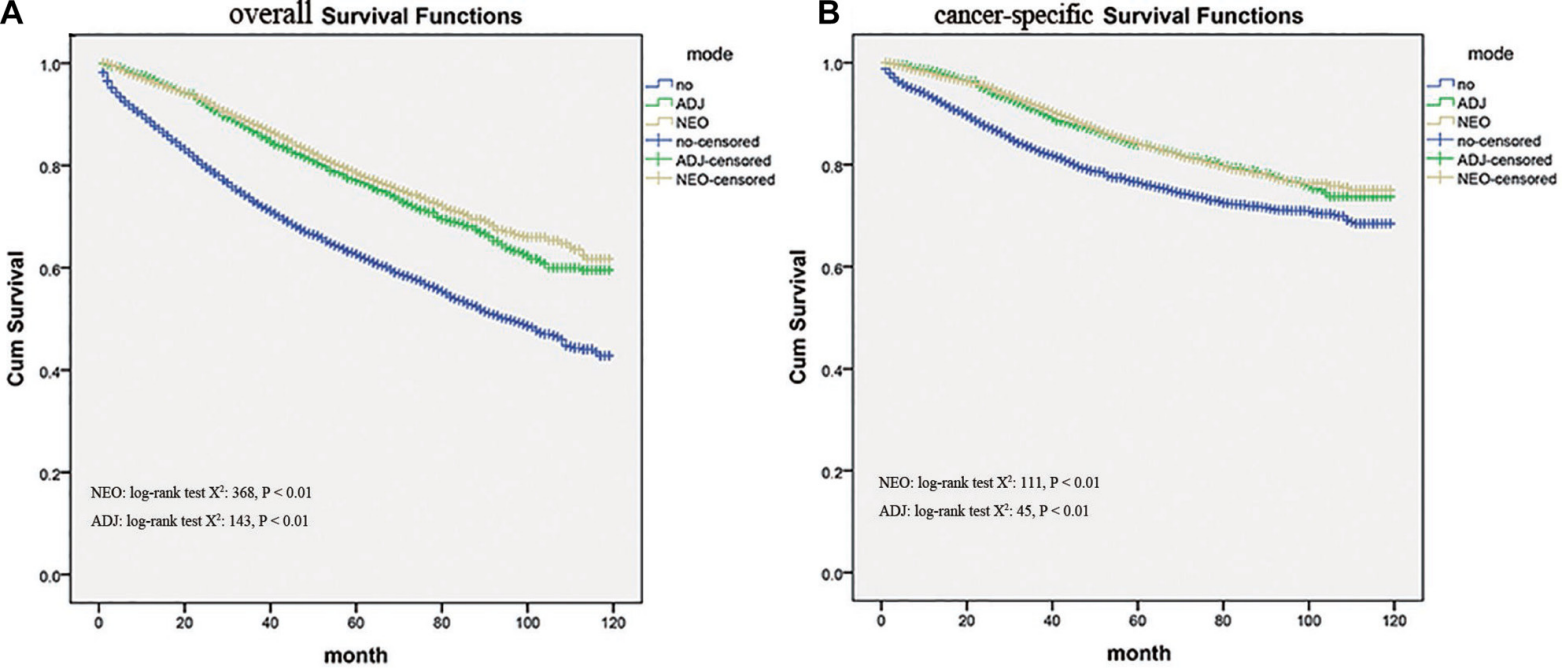
Kaplan–Meier survival curves showing overall survival and cause-specific survival (CSS) differences among Neo-Adjuvant radiation (NEO), Adjuvant radiation (ADJ), and No radiation (NO) cases

It was revealed in Kaplan-Meier survival analysis that those undergoing neo-adjuvant or adjuvant radiotherapy had better overall survival compared with those who did not. (log-rank test, *P* < 0.01) (Figure 1A). And the overall survival curves were similar between neo-adjuvant and adjuvant radiation groups by nonparametric. Moreover, Kaplan-Meier curves for rectal cancer-specific survival diverged with statistically significant differences in outcomes based on radiation therapy use (*P* < 0.01, Figure 1B). Unadjusted hazard ratios (HRs) were calculated based on neo- and adjuvant radiation status with crude HRs of 0.606 (95% CI, 0.552–0.666) and 0.646 (95% CI, 0.571–0.730) for cancer-specific survival.

After adjusting for possible confounders, statistically significant difference in cancer-specific survival was also seen for neo-adjuvant and adjuvant radiation with adjusted HR 0 .814 (95% CI, 0.722–0.918) and HR 0.848 (95% CI, 0.737–0.976).

### Multivariable analysis

It was confirmed in multivariable analysis that receiving any additional radiation therapy was associated with improved survival, and there was no difference in survival benefit between neo- and adjuvant radiation group. Besides, patients with 15 or more retrieved regional lymph nodes had significantly better survival than those with less than 15 nodes, and female gender was also an independent predictor of better survival. On the contrary, increasing age, poor differentiated histology and large tumor size seemed to confer worse survival effect with statistical significance (Table 4). That is why we are wondering whether there is any effect conferred by these confounders to survival benefit of radiation.

**Table 4:** Multivariable cox- proportional hazards survival analysis

Variables	Hazard ratio	95% CI	*P* value
**RT pattern**			
** No RT**	1 (reference)		
** Adjuvant**	0.848	0.737–0.976	0.021
** Neo-Adjuvant**	0.814	0.722–0.918	0.001
**Gender**			
** Male**	1 (reference)		
** Female**	0.863	0.779–0.956	0.005
**Age, Y**			
** < 65**	1 (reference)		
** 65–84**	1.668	1.498–1.859	< 0.01
** > 85**	3.961	3.346–4.690	< 0.01
**Race**			
** White**	1 (reference)		
** Black**	0.83	0.42–1.55	0.86
**Other**	1.02	0.98–1.86	0.74
**Marital status**			
** Married/partnered**	1 (reference)		
** Un-partnered**	1.56	0.95–2.43	0.63
**Grade**			
** well**	1 (reference)		
** moderate**	1.024	0.847–1.238	0.807
** poor**	1.501	1.202–1.875	< 0.01
** undifferentiated**	1.804	1.097–2.966	.020
**Tumor size (mm)**			
** < 50**	1 (reference)		
** 50–100**	1.249	1.121–1.391	< 0.01
** >100**	1.876	1.351–2.606	< 0.01
**Lymph node examined**			
** < 15**	1 (reference)		
** ≥ 15**	0.545	0.484–0.614	< 0.01
**Surgical type**			
** Sphincter preservation**	1 (reference)		
** APR**	0.92	0.984–1.614	0.56

RT indicates radiotherapy; APR, Abdominoperineal resection; Y, year.

### Sub-analysis of younger and older age

**Figure 2:**
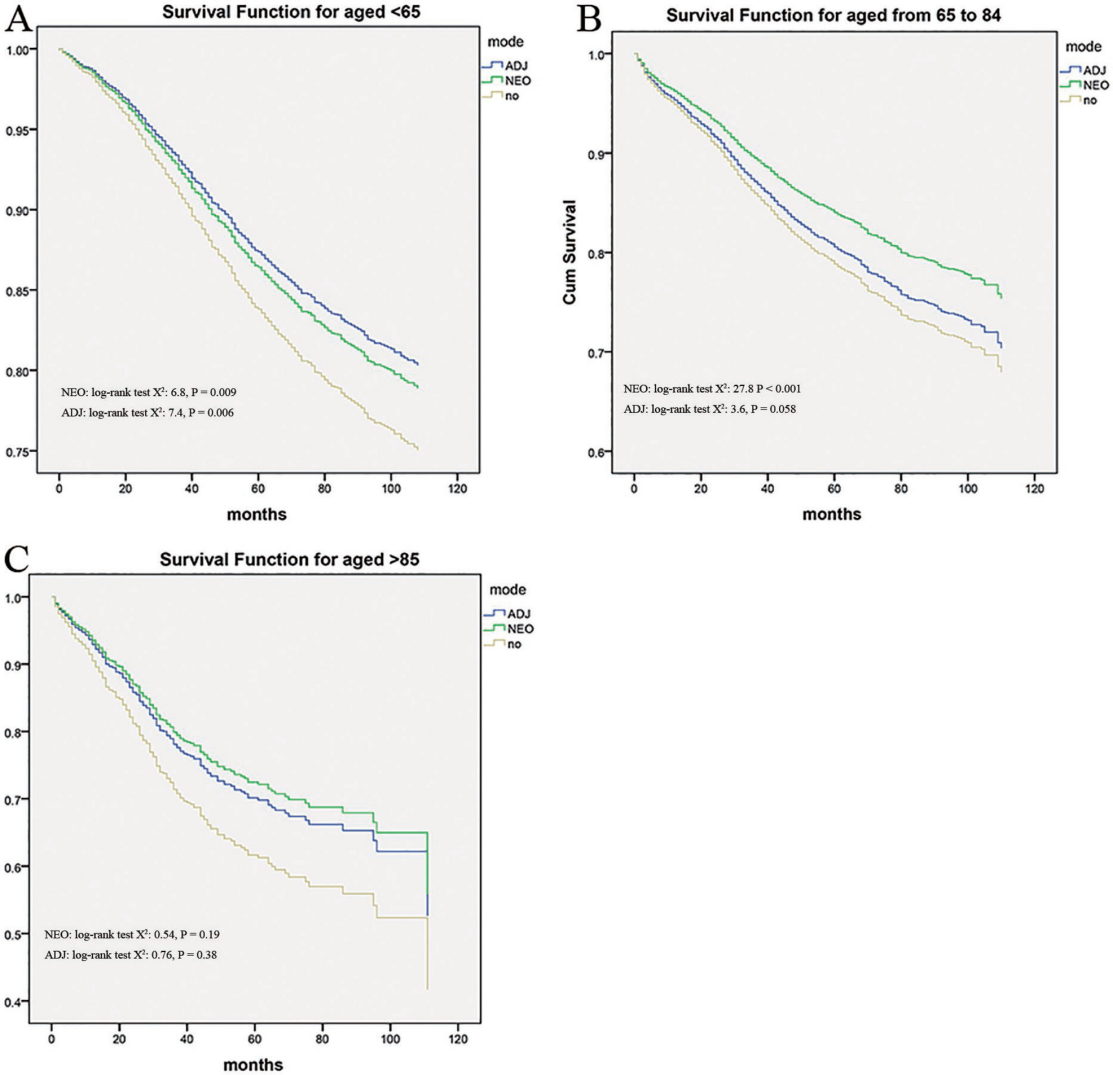
Impact of age on the cause-specific survival among Neo-Adjuvant radiation (NEO), Adjuvant radiation (ADJ), and No radiation (NO) cases

We also determined the impact of age on the efficacy of radiation therapy among patients in stage IIA. Modeling age as a categorical variable (age < 65 years, age ranged from 65 to 84 years and > 85 years), there was a significant interaction between age and efficacy of radiation therapy in terms of cancer-specific survival. Unsurprisingly, the addition of neo-adjuvant and adjuvant radiotherapy improved cancer-specific survival compared to no any radiation in patients younger than 65 years (HR = 0 .803, 95% CI, 0.662–0.974, and HR = 0.726, 95% CI, 0.624–0.844, Figure 2A ), but adjuvant radiotherapy seemed not to provide the beneficial effect ( HR = 0 .974, 95% CI, 0.823–1.153) just as the neo-adjuvant radiation therapy did ( HR = 0.7, 95% CI, 0.607- 0.807 ) in patients aged from 65 to 84 years. See Figure 2B. In the end, it was surprised that there was no obvious benefit among patients aged ≥ 85 years (*P* = 0.297 and 0.101, see Figure 2C), no matter which pattern of radiation was applied.

### Sub-analysis of tumor size

**Figure 3:**
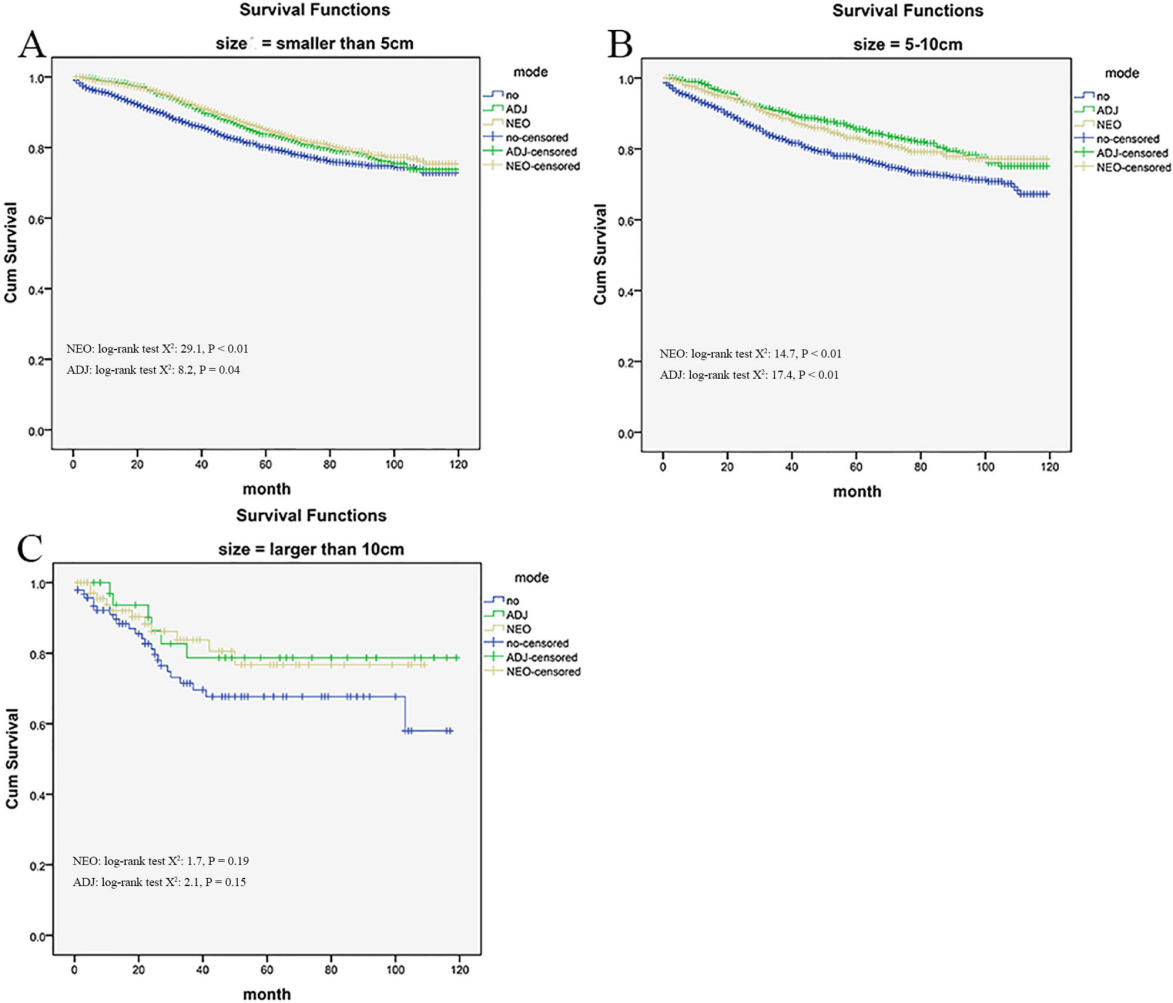
Impact of tumor size on the cause-specific survival among Neo-Adjuvant radiation (NEO), Adjuvant radiation (ADJ), and No radiation (NO) cases

Kaplan–Meier unadjusted survival analysis stratified by tumor size (< 50 mm, ranged from 50 to 100 mm and ≥ 100 mm) was used to compare survival among no radiation group, the neo- adjuvant and adjuvant radiation group. Then the better outcome was produced from radiation (all *P* < 0.05, see Figure 3A and Figure 3B) relative to no radiation, when the tumor size was smaller than 100 mm. But cohorts with larger than 100 mm in tumor size were generated for survival analysis, and the survival benefit conferred by neo-adjuvant and adjuvant radiation therapy was not observed, with comparable outcome among groups (Log-Rank x^2^ test: 1.722, *P* = 0.189 and Log-Rank x^2^ test: 2.043, *P* = 0.153, Figure 3C).

### Sub-analysis of retrieved regional lymph nodes

**Figure 4:**
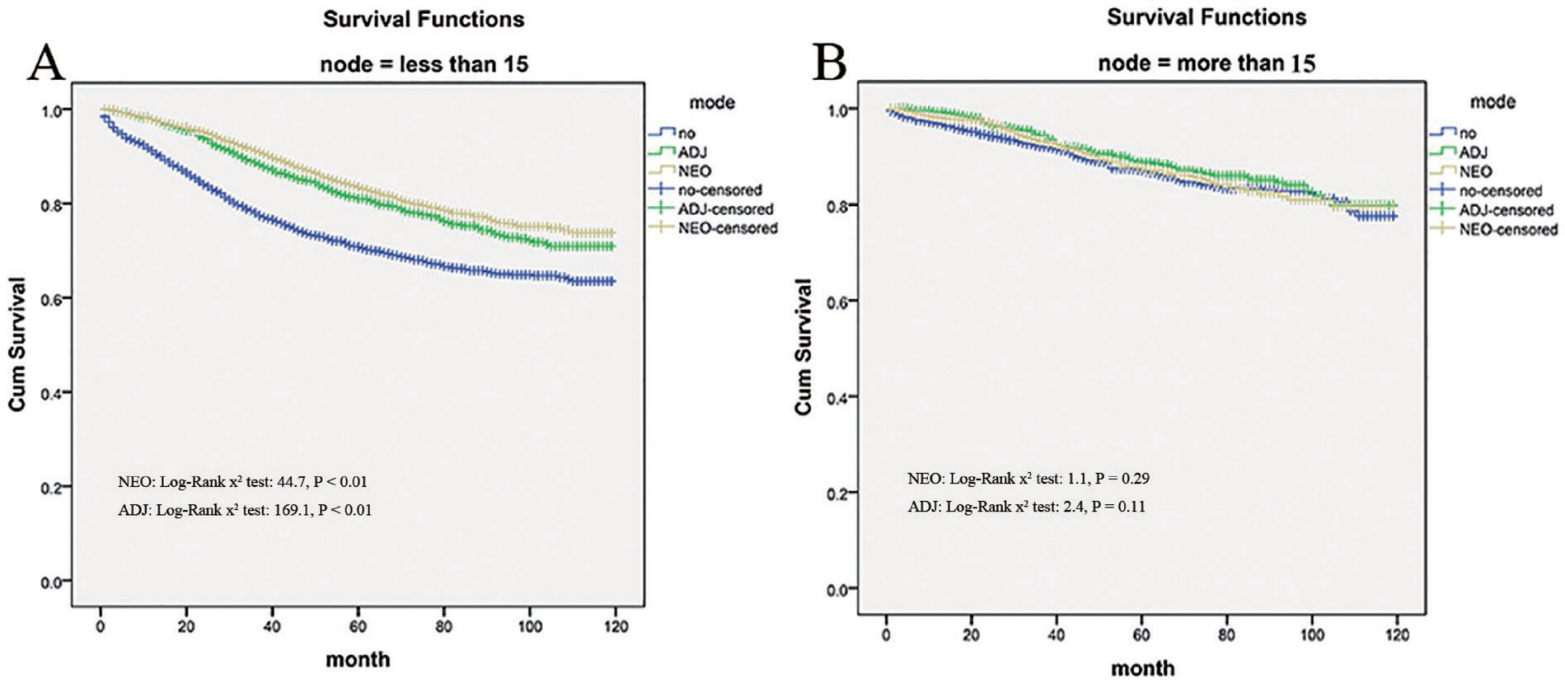
Impact of retrieved regional lymph nodes on the cause-specific survival among Neo-Adjuvant radiation (NEO), Adjuvant radiation (ADJ), and No radiation (NO) cases

For patients with 1 to 15 involved regional lymph nodes, neo-adjuvant and adjuvant radiation therapy provided significantly better survival than no radiation therapy(Log-Rank x^2^ test: 44.76, *P* < 0.01 and Log-Rank x^2^ test: 169.12, *P* < 0.01, Figure 4A). Yet radiation use was not associated with reduced mortality for patients with 15 or more involved nodes (*p* = 0.292, Figure 4B).

### Radiation effects stratified by histological grade

**Figure 5:**
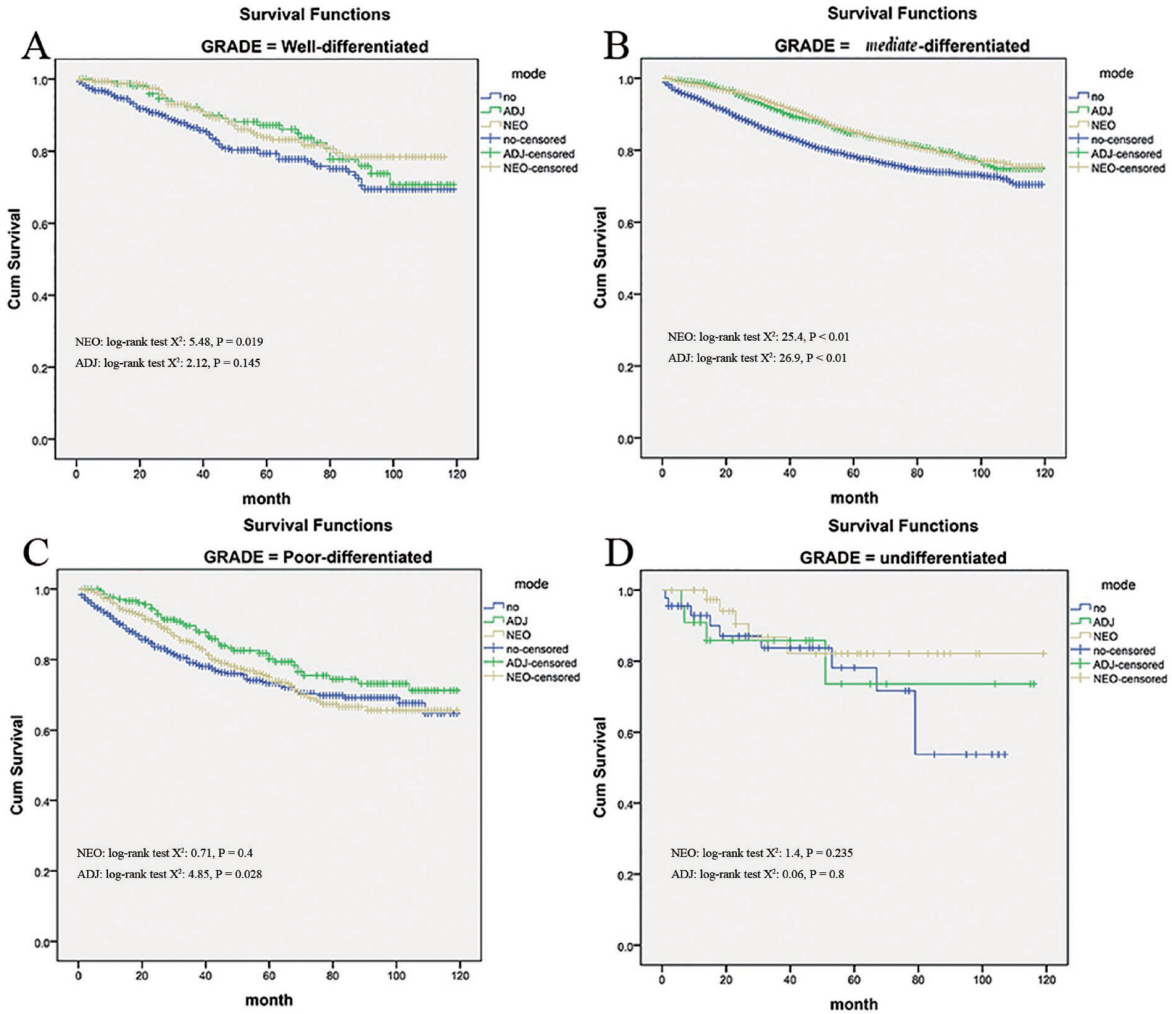
Impact of histological grade on the cause-specific survival among Neo-Adjuvant radiation (NEO), Adjuvant radiation (ADJ), and No radiation (NO) cases

Based on the histological grade of rectal cancer, radiation therapy did not seem to yield the same survival benefit effects. Although in the subset of moderate-differentiated tumors, neo- and adjuvant therapy were all associated with reduced mortality (all *P* < 0.01, Figure 5B) comparing to no radiation group. For well-differentiated tumors, the benefit effects were only conferred by the neo-adjuvant radiation therapy (Log-Rank x^2^ test: 5.48, *P* = 0.019) rather than adjuvant radiation therapy (Log-Rank x^2^ test: 2.126, *P* = 0.145, Figure 5A). In addition, just adjuvant radiation therapy had a positive impact (Log-Rank x^2^ test: 4.85, *P* = .028) upon survival in patients with poor differentiation, while, neo-adjuvant radiation therapy has not (Log-Rank x^2^ test: 0.71, *P* = 0.4, Figure 5C). However, it should be noted that cancer-specific survival within the undifferentiated tumors was analyzed and no difference was found in patients with radiation or not (*P* = 0.8 and *P* = 0.235, Figure 5D).

### Radiation effects stratified by gender

**Figure 6:**
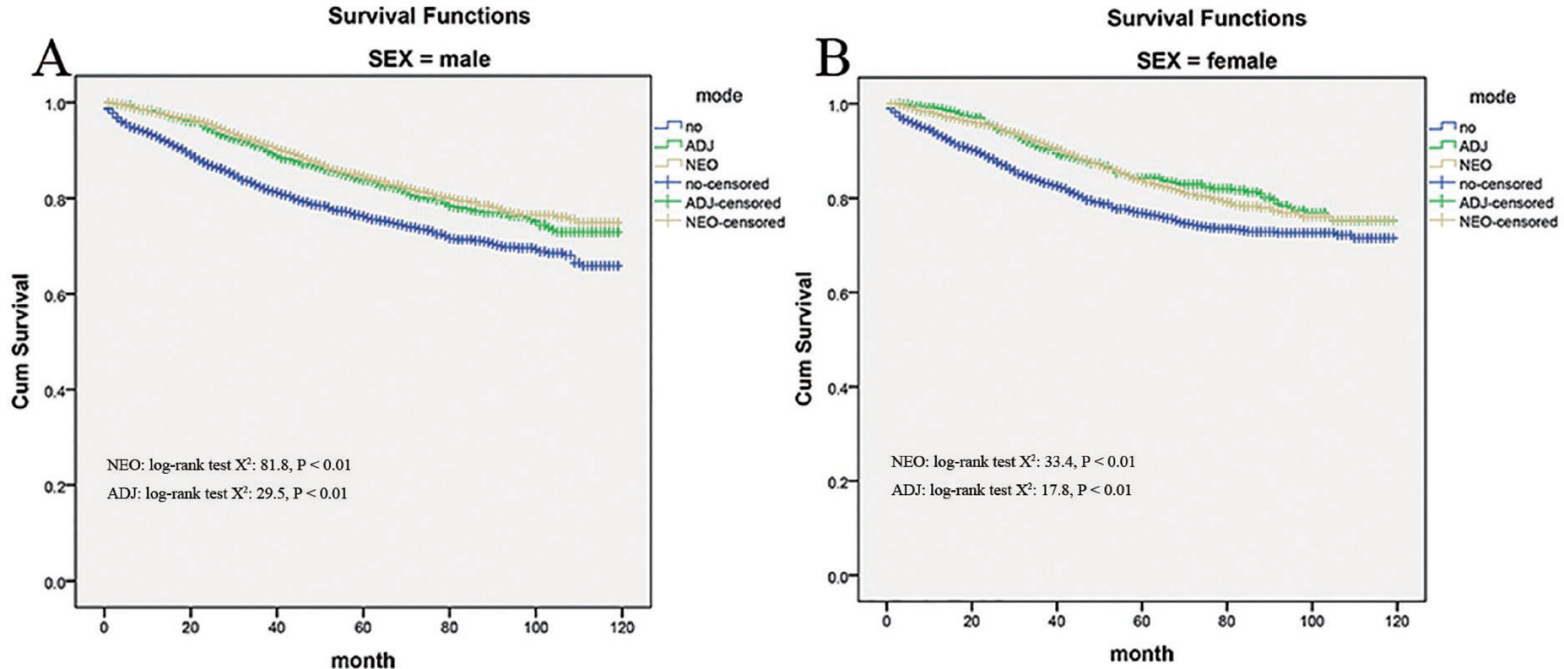
Impact of gender on the cause-specific survival among Neo-Adjuvant radiation (NEO), Adjuvant radiation (ADJ), and No radiation (NO) cases

For male patients with stage IIA rectal cancer, there was improved cancer-specific survival for those who received neo- and adjuvant therapy compared with those who did not (mean survival months: 102.3 and 101.1 versus 93.2 respectively; *P* < 0.001, Figure 6A). And in female patients, a similar survival benefit was noted for neo-adjuvant and adjuvant therapy compared to no radiation group (mean survival months: 102.0 and 102.8 versus 95.2 respectively; *P* < 0.001, Figure 6B). It did not appear that gender could affect survival benefit conferred by radiation therapy.

### Factors affecting the odds of having RT

On the one hand, there was a trend towards receiving additional radiation therapy in male in the adjusted analysis (odds ratio: 1.31, 95 % CI, 1.206–1.422; *P* < 0.001), and the rate of additional radiation therapy increased significantly when the grade of histology was shown as poor differentiation and un-differentiation. On the other hand, increasing age, relative large tumor size, and more retrieved regional lymph nodes were predictive of receiving no additional radiation therapy on multivariable analysis (Table 5).

**Table 5:** Factors affecting the odds of having radiotherapy

Variables	Odd ratio	95% CI	*P* value
**Gender**			
** Female**	1 (reference)		
** Male**	1.31	1.206–1.422	< 0.01
**Age, Y**			
** < 65**	1 (reference)		
** 65–84**	0.489	0.450–.532	< 0.01
** > 85**	0.157	0.129–.190	< 0.01
**Race**			
** White**	1 (reference)		
** Black**	0.76	0.53–1.25	0.66
** Other**	1.52	0.58–2.36	0.64
**Marital status**			
** Married/partnered**	1 (reference)		
** Un-partnered**	1.02	0.47–2.13	0.73
**Grade**			
** well**	1 (reference)		
** moderate**	0.986	0.846–1.150	.857
** poor**	1.066	0.878–1.294	.517
** undifferentiated**	1.292	0.826–2.022	.262
**Tumor size (mm)**			
** < 50**	1 (reference)		
** 50–100**	0.691	0.632–.755	< 0.01
** > 100**	0.995	0.731–1.354	.975
**Lymph node examined**			
** < 15**	1 (reference)		
** ≥ 15**	0.443	0.406–0.482	< 0.01

Y indicates year.

## DISCUSSION

In this population setting, it appeared that patients with stage IIA rectal cancer treated without radiation therapy experienced a significantly worse overall survival and cancer-specific survival compared to patients with neo-adjuvant and adjuvant radiation therapy. However, radiation therapy did not seem to yield the same survival benefit on the basis of histological grade of rectal cancer, age, retrieved regional lymph nodes and tumor size.

The rationale of radiation therapy stems from high-risk patients undergoing surgery for advanced rectal cancer. The goal of utilizing additional radiation therapy in rectal cancer is straightforward: to prevent locoregional relapse along with its linked morbidity and mortality. It has been confirmed that improved local control and survival have been achieved with the addition of radiation therapy in management of patients with resectable rectal cancer. As the previous evaluation in unselected rectal cancer, additional radiotherapy produced much lower local relapse rates to 10% and improved survival rates of 60%, compared with the results of up to 25% local failure rate and 40%–50% long-term survival produced by surgery alone [6, 13]. Although there existed much less risk of disease recurrence and mortality in patients with pathologic stage IIA rectal cancer, in this study, we also can see improved survival rates as 75.1% and 73.8% in neo-adjuvant and adjuvant radiation group, compared with 68.4% in patients receiving no radiotherapy in terms of 10-year cancer specific survival. A partial component of this survival benefit can be explained by the disagreement found between clinical and pathological nodal staging in some stage IIA patients. It was assumed that 28% of patients with rectal cancer clinically staged as IIA were identified to have lymph node metastases at surgical pathology [14]. Even through strictly either EUS or MRI staging, 22% of patients assumed as negative lymph nodes rectal cancer have been proved pathologically positive lymph nodes [15]. In another word, the low accuracy of available diagnostic tools in identifying metastatic nodes does not allow us to perform tailored treatments in patients with clinically staged IIA rectal cancer, thus, additional radiotherapy remains the care standard for locally stage II rectal cancer.

In fact, our results also indicate an essential role of radiation therapy in the management of accurately pretreatment identified stage IIA cancer, and it was consistent with the evaluation by Kennecke H [5] who found that unselected patients with stage IIA rectal cancer not treated with radiation experienced reduced disease-specific survival in comparison to patients treated with additional radiation therapies. The survival benefit by radiation may stemmed from the relapse control, since an early and sustained lower locoregional relapse rates were demonstrated in radiation group as 6.3% comparing to 29% for the no radiation group. Disease-specific survival was numerically superior in radiation therapy relative to no radiation patients, nevertheless this result did not achieve statistical significance (HR = 0.62 [0.33–1.17]). Even a cohort study [9] (*n* = 151) concluded that additional postoperative radiotherapy did not alter local recurrence or survival after radical surgery in patients with stage IIA rectal cancer. They indicated postoperative radiation may be an overtreatment as an adjuvant therapy in patients with stage IIA rectal cancer if they had no other risk factors. One possible explanation for this conflicting result is that different pattern of radiation therapy did not seem to yield the same survival effects in stage IIA rectal cancer. Just as the NSABP randomized trial [7], it was found neo-adjuvant radiation therapy offered a significantly improved 5-year disease free survival (65% vs. 53%; *P* = 0.011) when compared with adjuvant radiation. Meanwhile, it was reported that preoperative radiotherapy, as compared with postoperative radiotherapy, improved local control and was associated with reduced toxicity [2]. Moreover, neo-adjuvant radiation therapy was associated with significantly decreased 5-year local relapse (5.8 vs. 19.4%; *p* = 0.02) and increased OS (88.4 vs. 65.7%; *p* = 0.001), in comparison with post-operative radiation in unselected rectal cancers [16]. In line with the sub-analysis of this study, for well -differentiated tumors, the benefit effects were only conferred by the neo-adjuvant radiation therapy (Log-Rank x^2^ test: 5.48, *P* = 0.019) rather than adjuvant radiation therapy (Log-Rank x^2^ test: 2.126, *P* = 0.145). it was speculated that a major component of the pre-operative radiation benefit over post-operative RT may simply reflect better compliance, and there was a hypotheses that launching systemic therapy as early as possible might treat systemic micro-metastases more effectively, as well as better tolerance expected.

One important potential confounder in the evaluation of radiation treatment is that older age at diagnosis was found to significantly influence the efficacy of radiation therapy in treating stage II disease, and patients without radiation tended to be older (mean age of 68) compared to adjuvant RT (mean 61.73) or Neo-adjuvant RT (mean 61.09) patients. We can indentify that there was a significant interaction between age and efficacy of radiation therapy in terms of cancer-specific survival. In the middle age, the addition of neo-adjuvant and adjuvant radiation therapy improved cancer-specific survival compared with no radiation, while, in patients aged from 65 to 84 years, only the neo-adjuvant radiation therapy was able to provide the beneficial effect rather than adjuvant radiation therapy. In the end, there was no obvious benefit among patients aged ≥ 85 years in any radiation treatment. In general, younger age and lower co-morbidity score were all associated with addition of adjunctive therapy, such as radiation [17], exactly, radiation-induced complications have been clearly found more frequently in the elderly. The rate of acute toxicities was assumed to be elevated up to 48% after radiotherapy for rectal cancer, and 10% of patients experienced serious toxicities requiring hospitalization or surgical intervention, resulting in a poor quality of life even affecting oncologic outcomes [18]. Based on radiation-related toxicities, some patients receiving radiotherapy may be disadvantageous in terms of poor outcome, particularly in stage IIA disease. Given the potential disadvantages shown in this study, it is essential to ensure that elderly patients should have adequate supports and counseling regarding the benefits, risks, and side effects of additional radiation.

It is recommended that at least 12 lymph nodes harvest in the specimen should be assured to avoid understaging [19]. It is adequate as a reflection of surgical quality and precisely assessment of colorectal staging. Nevertheless, the outcome for TNM stage II patients remains highly variable, even with lymph node harvests in standards. A proportion of this disappointing outcome may be explained by heterogeneous response to adjuvant therapy, like radiation. In this study, for patients with 1 to 15 regional lymph nodes harvests, neo-adjuvant and adjuvant radiotherapy provided significantly better survival than no radiation therapy (all *P* < 0.01), yet radiation use was not associated with reduced mortality for patients with 15 or more involved nodes (*p* = 0.292). Why does the efficacy of radiotherapy depend on the number of lymph nodes examined? The differing responses of the same stage patients enrolled in this subject could be attributed to differences risk for recurrence and survival in different lymph nodes harvest and only high-risk stage IIA cancer can be beneficial from additional radiotherapy. Since in TNM stage II patients, it demonstrated that the 10-year survival rates were clearly dependent on the number of lymph nodes examined. In addition, compared to cases with more than 19 nodes, the examination of less than 10 nodes increased a patient’s hazard to 1.501. Hence, it was assumed that the number of lymph nodes examined had an effect on prognosis of stage II colorectal cancer, indicating fewer retrieved nodes as a powerful risk factors for recurrence and survival [20]. It is possible that less immune response was generated by aggressive tumors, or else the patients with fewer lymph nodes harvests may be associated with diminished immune response, which can lead to smaller lymph nodes, as well as a lower number being identified [21]. In line with this hypothesis that a poorer prognosis was implicated by a small number of lymph nodes examined because of an insufficient immune response, there was an observation that the pattern of failure in stage II CRC patients with low lymph node counts is mainly characterized by an increased distant metastatic disease, especially hepatic metastases [22]. Above all, in the light of the above implication, we have established the number of lymph nodes harvest as a specific biologic marker in detecting high-risk patients and improving their prognosis with additional radiation therapy tailored.

Although SEER is characterized by a large sample of patients for identifying gross trends and interesting patterns, there also existed some important limitations such as ours. First of all, reporting bias is inherent in any retrospective database, so appropriate adjustment for potential confounders is performed to determine the effect of an intervention. Secondly, the data set also does not include certain important prognostic indicators that could confound the survival analyses, such as type and course of chemotherapy or dose and fractionation of radiation. In addition, given the risks of radiation therapy, unmeasured confounders, most importantly comorbid illness, should be accounted for in the data.

In conclusion, we infer that stage IIA rectal cancer patients receiving radiotherapy with neo- or adjuvant pattern had additional benefit in terms of overall and cancer-specific survival. However, radiation therapy did not seem to yield the same survival benefit, moreover, histological grade of rectal cancer, age, retrieved regional lymph nodes and tumor size were the potential influential prognostic parameter. Finally, the decision to offer radiation for such patients must be individualized based on clinicopathologic risk factors and patient preference.

## MATERIALS AND METHODS

To assess patterns of utilization and the effect of additional radiation therapy, this cohort study was performed on the patients with first primary rectal cancer extracted from the 1988 to 2013 in Surveillance, Epidemiology and End Results (SEER) program database. The SEER data on cancer trends included multiple population-specific cancer registries across the U.S., and gave rise to a largest volume of cancer-related data and statistics [12]. All patients with stage IIA tumors, defined as depth of invasion beyond the outer border of the muscular layer, and no nodal involvement were enrolled in the current analysis according to the American Joint Committee on Cancer staging system. The pattern treatment sequence consisted of ‘‘radiation before surgery’’ (Neo-Adjuvant RT), ‘‘radiation after surgery’’ (Adjuvant RT), or ‘‘no radiation and/or cancer directed surgery’’. Following the National Institutes of Health Consensus Conference was claimed by SEER database in terms of treatment. Due to limited quantity, it was excluded when intraoperative or perioperative radiation therapy was performed. In addition, patients died within 30 days after surgery were not included.

### Outcome variable and covariates

The primary outcome variable for our study was survival time. The overall survival and rectal cancer-specific survival were both evaluated in the present study. They were censored who died of other causes or were still alive at the end of the follow period. As the possible confounding factors, we also retrieved gender, age at diagnosis, race, histological grade, tumor size, regional nodes examined, marital status, radiation type, cause-specific death classification, survival time, and vital status information.

### Statistical analysis

Descriptive and survival statistical analyses were used to evaluate the radiation therapy and the impact of this adjunct to surgical treatment of rectal cancer on patient outcomes. Categorized data were summarized using contingency tables and assessed by chi-square test or Fisher exact test for demographic and tumor characteristics of patients from these 3 groups (No RT, Adjuvant RT, and Neo-adjuvant RT). Our analyses were adjusted for age, race, year of diagnosis, primary tumor size, histological grade, number of lymph nodes examined, type of radiation and marital status. And we also performed multivariate Cox proportional hazards regressions with Adjuvant RT, Neo-Adjuvant RT, histological grade, tumor size, year of diagnosis, and demographic characteristics as covariates. Survival outcomes including overall and cancer specific survival were calculated to evaluate the impact of radiation therapy using Kaplan-Meier analysis. Patients with additional radiation therapy were compared to those without radiation therapy. The log-rank test was used to determine whether the Kaplan-Meier survival curves for the two groups were statistically equivalent. Above all, cut points for categorical variables including age, tumor size, and number of sampled nodes were chosen to identify the effect of radiation in sub-analysis, which allowed for estimation of the treatment effect on survival while minimizing bias. A *P* value of <0.05 was considered to reach statistical significance. Statistical analyses were performed using the statistical software package SPSS for Windows, version 22 (IBM SPSS Inc, Chicago, IL).
